# Subjective Experience of Episodic Memory and Metacognition: A Neurodevelopmental Approach

**DOI:** 10.3389/fnbeh.2013.00212

**Published:** 2013-12-25

**Authors:** Céline Souchay, Bérengère Guillery-Girard, Katalin Pauly-Takacs, Dominika Zofia Wojcik, Francis Eustache

**Affiliations:** ^1^LEAD UMR CNRS 5022, Université de Bourgogne, Dijon, France; ^2^Department of Experimental Psychology, University of Bristol, Bristol, UK; ^3^U1077, INSERM, Caen, France; ^4^UMR-S1077, Université de Caen Basse-Normandie, Caen, France; ^5^UMR-S1077, Ecole Pratique des Hautes Etudes, Caen, France; ^6^UMR-S1077, CHU de Caen, Caen, France; ^7^School of Social, Psychological and Communication Sciences, Leeds Metropolitan University, Leeds, UK; ^8^Department of Psychology, University of Valladolid, Valladolid, Spain

**Keywords:** episodic memory, recollection, metamemory, neurodevelopmental disorders

## Abstract

Episodic retrieval is characterized by the subjective experience of remembering. This experience enables the co-ordination of memory retrieval processes and can be acted on metacognitively. In successful retrieval, the feeling of remembering may be accompanied by recall of important contextual information. On the other hand, when people fail (or struggle) to retrieve information, other feelings, thoughts, and information may come to mind. In this review, we examine the subjective and metacognitive basis of episodic memory function from a neurodevelopmental perspective, looking at recollection paradigms (such as source memory, and the report of recollective experience) and metacognitive paradigms such as the feeling of knowing). We start by considering healthy development, and provide a brief review of the development of episodic memory, with a particular focus on the ability of children to report first-person experiences of remembering. We then consider neurodevelopmental disorders (NDDs) such as amnesia acquired in infancy, autism, Williams syndrome, Down syndrome, or 22q11.2 deletion syndrome. This review shows that different episodic processes develop at different rates, and that across a broad set of different NDDs there are various types of episodic memory impairment, each with possibly a different character. This literature is in agreement with the idea that episodic memory is a multifaceted process.

## Introduction

Episodic memory is a system which permits people to retrieve memories characterized by temporal, spatial, and self-referential features. Tulving (1985) characterized episodic memory as “autonoetic” (self-knowing). This state of awareness was related to the recollection of a specific personal context for the retrieved information. From this original description, many different theories have spawned (see reviews from Yonelinas, 2002; Mandler, 2008; Moulin et al., 2013). In experimental settings, recollection is often operationalized as the “recall of information that was experienced during the study episode that is cued by a recognition test stimulus” (Montaldi and Mayes, 2010, p. 1294), and can either be measured by subjective report (e.g., Tulving, 1985) or by objective reports asking the participant to reproduce material from the study phase, such as the source of the information (e.g., DeMaster and Ghetti, 2013). It can be characterized as the retrieval of “something more” – the idea that associated thoughts, feelings, or material from the time of encoding come to mind or can be brought to mind at the time of retrieval (Moulin et al., 2013).

The idea of recollection places great emphasis on operations occurring at retrieval, rather than the content or “type” of memory retrieved (Klein, 2013). Klein et al. (2004) suggested that for memory content to be experienced as episodic at retrieval there are four critical factors: (1) sense of agency, (2) sense of ownership, (3) a capacity for self-reflection, and (4) a sense of time as personal events happening in relation to the self. Such a capacity for reflection is defined as “metacognitive” – the ability to know about one’s own mental states. A prominent model of metacognition (Nelson and Narens, 1990) involves two levels of cognitive processes, an exchange of information between a higher order representation and an object level, where mental operations are carried out. Memory proficiency is achieved by the regulation and awareness of the information exchanged between these two levels. In the case of recollection, presumably on-line feelings and thoughts generated during retrieval by the object level are monitored by the meta-level, leading to the implementation of mnemonic strategies, termination of search, and so on.

Retrieval from episodic memory, then, is a complex reflective process. There is a an extremely large literature which points to the complexity of the episodic system, and in healthy adults, a lot is known about the strategic and reflective processes which contribute to effective memory function. Much of this literature considers metamemory and subjective report. The aim of this review is to set this large literature against what we currently know about episodic memory in children and neurodevelopmental disorders (NDDs). We focus on episodic memory as measured by the recall of rich contextual details and in recollection paradigms (described below). We also present subjective states as measured by first-person experiences of remembering. Finally, to consider strategic and reflective aspects to episodic memory, we consider metamemory function. We start by reviewing healthy development (see Typically Developing Children). The last part of the paper then considers episodic memory, metamemory, and subjective states of episodic memory in NDDs with a neuropsychological approach (see Neurodevelopmental Disorders). In the discussion (see Discussion), we show that this review supports the idea of a fractionation of the episodic memory system and how a developmental approach agree with the idea that there are separable subjective and objective components in episodic memory.

## Typically Developing Children

Many studies have now explored children’s ability to remember detailed memories and whether this capacity improves throughout childhood and adolescence (see Episodic Memory below). In contrast, the development of the ability to introspect on memory contents (subjective states of recollection) or estimate memory contents (metamemory) has not been explored so often. Because one of the main issues in the subjective experience of memory is the putative difference between recollection and familiarity, we next review these two processes in healthy children (see The Subjective Experience of Memory below). We then develop this emphasis on subjective experiences to take on well-known measures of retrieval failure and metacognitive paradigms such as the feeling of knowing (FOK) (see Metamemory below). Finally, we provide some neuroimaging findings to illustrate the contribution of the brain maturation to the development of episodic memory (see Neurodevelopmental Approach below).

### Episodic memory

Tulving (2002) suggested that the ability to form episodic memories does not emerge until 4 or 5 years of age (Newcombe et al., 2007; Hayne and Imuta, 2011; Scarf et al., 2013). In fact, several studies show that the ability to form new specific personal events, rich in contextual details, improves during childhood until adolescence (e.g., Brainerd et al., 2004; Ghetti and Angelini, 2008; Howe et al., 2009). For example, Bauer et al. (2012) showed an age-related difference in the development of children’s memory for the spatial locations of personal events; older children are more likely to integrate spatial information into their autobiographical memories than younger children.

Many studies now suggest the existence of different developmental trajectories for the different components of episodic memory. In particular, recent studies have shown that familiarity-based processes develop earlier than recollection-based ones (Billingsley et al., 2002; Ghetti and Angelini, 2008; Brainerd et al., 2012). Research has also shown that recalling contextual information develops later than recalling the information itself (Cycowicz et al., 2001, 2003; Pirogovsky et al., 2006). In this context, Sluzenski et al. (2006) showed an age-related improvement in remembering the association between contextual information and factual content between the age of 4 and 6 (see Picard et al., 2012 for similar findings).

### The subjective experience of memory

Studies exploring developmental changes in the ability to introspect on memory are relatively limited. Research into subjective experience of memory in children has mostly used the Remember/Know paradigm (RK, Tulving, 1985). This requires participants to categorize their responses on a recognition memory test according to whether they *remember* the answer, as opposed to *knowing* it or finding it *familiar*. A first study by Perner and Ruffman (1995) suggested that young children could not reliably differentiate remembering and knowing; children could not judge what originates from personal experience before the age of 3. In a more recent study, Ghetti et al. (2011) also asked children to classify memories into “Remember” or “Familiar” categories. They showed that 6- to 7-year-olds found it difficult to differentiate between states of recollection and familiarity but their level of understanding was nonetheless above chance.

From about the age of 8, children begin to report more experiences of remembering on memory tests, with a developmental trend in the subjective experience of recollection (Billingsley et al., 2002; Ofen et al., 2007; Piolino et al., 2009; Friedman et al., 2010). For example, Billingsley et al. (2002) showed an increase in the proportion of Remember responses with age (from 8 to 19 years), with the youngest group giving fewer correct Remember responses (5% for the youngest group versus 28% for the oldest group). In a more recent study also using the same paradigm, Rhodes et al. (2011) confirmed these findings and suggested that 11-year-olds can engage recollection to the same degree as adults.

Ghetti et al. (2011) explored whether or not the nature of the subjective recollection changed during childhood, and whether the qualitative details of memories changed. In their Experiment 2, children were shown line drawings presented either in red or green. At encoding, they were asked to state aloud the color and were also asked a semantic question. At the retrieval stage, participants reported Remember or Know for each item. This was followed by an old-new recognition decision and a source discrimination judgment (color and question asked). Across all ages, children were more likely to give correct source information to Remember judgments, showing that even young children (e.g., 6- to 7-year-olds) could differentiate between Remember and Familiar judgments. However, young children (before the age of 10) were more likely to wrongly associate correct source with Familiar judgments. Furthermore, older children were more likely to experience subjective recollection when they remembered semantic information. According to Ghetti et al. (2011), this indicates that older children are more aware of the factors which give rise to a feeling of recollection and are more skilled at using that diagnostic information. In other words, children possibly become metacognitively more competent at using source information to support the recollection of a detailed memory. In the next section we follow up this idea, reviewing the scientific literature on metamemory in healthy children.

### Metamemory

Historically, metamemory has either been investigated from a developmental psychology perspective (see Flavell, 1979) or experimental memory viewpoint (Hart, 1965). The developmental literature has mainly focused on memory strategy and what children know about memory functioning whereas, experimental memory research has generated specific and reliable methods to measure people’s ability to introspect on their memory processes (Nelson and Narens, 1990). Recent work has taken these experimental paradigms into developmental populations, uniting these two otherwise disparate approaches to metamemory (for reviews, see Schneider and Lockl, 2002; Lyons and Ghetti, 2010). In this work, experimental paradigms are used in which children are asked to predict their future memory performance either while retrieving the information (e.g., FOK; Hart, 1965; Schacter, 1983; Sacher et al., 2009) or during learning (e.g., Judgment-of-learning, JOL; Arbuckle and Cuddy, 1969). The extent to which children have proficient metamemory is captured in their ability to accurately predict their performance.

In a typical JOL procedure, participants are presented with cue-target pairs and asked to make a JOL reflecting the likelihood that they will later recall the target word when presented with the cue word. Using such a task, Koriat and Shitzer-Reichert (2002) showed a developmental trend, with children becoming more accurate as they get older and thus more able to predict their recall performance. In a typical episodic FOK experiment, participants are presented with word pairs and at test, if they cannot recall the target, they are asked to predict whether they will be able to recognize it later. FOK judgments are thus predictions about the likelihood of subsequent recognition of currently non-recalled information (Hart, 1965; Nelson and Narens, 1990). Using this paradigm, Wojcik et al. (2013a) have showed that children (aged 12) could accurately predict their memory performance when asked to give episodic FOK judgments. However, whether or not a development trend on episodic FOK exists is difficult to determine as most developmental FOK studies have used general knowledge, or pre-learned semantic material. However, such studies do report that FOK accuracy improves continuously across childhood and adolescence (Wellman, 1977; Cultice et al., 1983; Wojcik et al., 2013a, but, see Butterfield et al., 1988).

Other studies exploring the development of metacognitive monitoring have shown that the accuracy of confidence judgments about memory retrieval of individual items improves during late childhood (age 7–12 years; von der Linden and Roebers, 2006; Ghetti et al., 2008; Krebs and Roebers, 2010). Furthermore, young children have a good metacognitive knowledge (Flavell, 1979). Studies have also shown that from a young age children understand some of the factors influencing memory, thus showing metacognitive knowledge (Lyon and Flavell, 1994). For example, Ghetti et al. (2002) showed that children as young as 5 or 6 can monitor varying degrees of memory strength as indicated by changes in their confidence ratings. In a similar vein, two studies have demonstrated that children can accurately use their metacognitive knowledge to make predictions; children assign higher JOLs to judged-easy pairs than to judged-difficult pairs (Koriat and Shitzer-Reichert, 2002; Lockl and Schneider, 2003). Thus children can modulate their JOLs according to their metacognitive knowledge – they are sensitive to the difficulty of to-be-learned material.

Metacognitive judgments such as JOLs or FOKs are important as they will impact on the learning processes and the memory strategies put into place. This is illustrated in the metamemory framework proposed by Nelson and Narens (1990) by the fact that monitoring (the subjective experience) and control processes (the behavior) operate in a feedback loop: through memory monitoring, we control our memory function and implement appropriate mnemonic strategies (the “monitoring affects control hypothesis,” Nelson and Leonesio, 1988). As a result, proficient metamemory functioning should ensure effective memory performance. To the best of our knowledge only one study has explored the relationship between monitoring and control from a developmental perspective. Lockl and Schneider (2003) showed that for both first and third graders, JOLs made during the first study trial predicted the amount of time that the children invested in each item in a subsequent self-paced study trial. This relationship between JOL and study time became stronger for third graders (age 9 years).

### Neurodevelopmental approach

**Figure 1:**
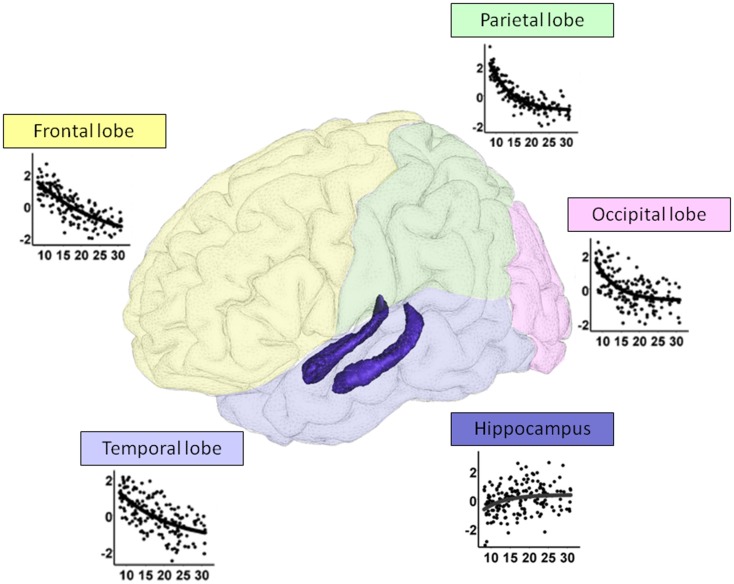
**Gray matter maturation from 8 to 30 years: regression plots showing the relationship between age and bilateral cortical volume (corrected for the total brain volume) of each lobe and the hippocampus**. The non-linear decrease in cortical lobe volume contrasts with a slight increase in hippocampal volume. Parietal and occipital lobe seem to mature earlier than temporal and frontal lobes. Adapted from Ostby et al. (2009).

Neuroimaging studies provide evidence that brain maturation contributes to the development of the episodic memory. There is a growing body of evidence showing that major age-related changes occur before puberty with a slight evolution until adulthood focusing mainly on frontal regions (Figure 1). These changes include cerebral networks devoted to memory functioning: medial temporal lobe structures, parietal, and frontal regions (Paz-Alonso et al., 2008; Ghetti et al., 2010). There is a complex developmental pattern observed in the connections between frontal and temporal regions, and development of these connections continues into adulthood. Otherwise, the only functional neuroimaging study in children which has focused on subjective recollection evaluated with the RK paradigm points to an age-related increase in activations of the prefrontal cortex (Ofen et al., 2007). Some other studies have revealed a medial temporal specialization with increasing age. Longitudinal studies showed that the anterior hippocampus may decrease relatively in volume from age 4 to 25, while the posterior hippocampus may increase (Gogtay et al., 2006). This structural maturation influences the cerebral network recruited to process episodic memory tasks with age as shown by functional brain imaging studies. For instance, Ghetti et al. (2010) were interested in activations during incidental encoding of items as a function of subsequent memory of items and details associated with target items. Their results revealed that youngest children, aged 8, recruit the hippocampus and posterior parahippocampal gyrus for both item recognition (line drawings) and associated details (color of ink), whereas the 14 years olds engaged these regions only for subsequent detail recollection. Finally, it is worth noting that the medial temporal and prefrontal regions are not the only structures implicated in the development of episodic memory. A graded activation of the posterior parietal cortex is associated with correct episodic performances (Paz-Alonso et al., 2008; Ofen et al., 2012; DeMaster and Ghetti, 2013).

A converging body of arguments also indicates that the posterior parietal cortex is implicated in episodic retrieval and subjective recollection in adults (Shimamura, 2011). The temporo-parietal junction a one of the cerebral regions referring to the Cortical Medial Structures (CMS) involved in autobiographical memory and critical for self-development (Pfeifer and Peake, 2012). The parietal cortex could also play an important role in mnemonic control by modulating top-down (frontal) and bottom-up (medial temporal structures) processes (Paz-Alonso et al., 2013). Hence, abnormal functioning of one cerebral region included into this network would result in a specific pattern of episodic impairment. This point will be detailed in the following sections.

## Neurodevelopmental Disorders

The cognitive neuropsychological approach to memory function has a long history of using patterns of deficit and dysfunction to better understand and treat memory impairment, but also patient studies yield an important data set by which to evaluate cognitive models and further theories. This review argues that a neuropsychological approach to episodic memory is necessary to understand further the development of episodic memory generally. In this section, studies on NDDs (such as autism or chromosome disorders) are presented to show how these disorders represent an opportunity to better understand the contributions of separate sub-components to episodic memory.

Neurodevelopmental disorders affect neural development with direct consequences on learning. A working definition was proposed by Milan (2013):

“NNDs are generally accepted to be disorders diagnosed before the age of 18 where: Central nervous system development is impaired and/or delayed, leading to either disruption of discrete cerebral functions, or to generalized impairment across multiple domains” (Milan, 2013, p. 8).

It is possible to further classify disorders according to whether they are genetic (either discrete genetic abnormalities, such as Down syndrome (DS); or multiple anomalies in “polygenic” disorders, such as autism), or acquired (early and congenital brain injuries, such as anoxia). In this review, our drive was to understand more about the processes involved in episodic memory function and dysfunction during development. Rather than being exhaustive in terms of etiology, we organize our review around the key populations which have attracted most attention: amnesia acquired in infancy following anoxia, traumatic brain injury (TBI) or tumors [see Amnesia Acquired in Infancy and Childhood (Anoxia, Traumatic Brain Injury, Brain Tumors)], autism (see Autism), and chromosome disorders (see Williams Syndrome, Down Syndrome, and 22q11.2 Deletion Syndrome).

### Amnesia acquired in infancy and childhood (anoxia, traumatic brain injury, brain tumors)

In this section, we present studies of children and adolescents with acquired amnesia due to anoxia, TBI, and brain tumors. Table 1 presents a summary of the different findings.

**Table 1:** **Key findings on episodic memory and metamemory in children and adolescents with anoxia, brain tumor, and traumatic brain injury**.

Article/etiology	Age (years)		Main finding
	At injury	At test	
**ANOXIA**			
Ostergaard (1987)	10 (single case)	15	Residual learning in the absence of measurable episodic memory
Broman et al. (1997)	8 (single case)	27	Severe episodic memory deficits
Vargha-Khadem et al. (1997)	Perinatal (single cases)	14, 19, 22	Profound episodic memory deficit in the context of normal knowledge acquisition during childhood
Baddeley et al. (2001), Düzel et al. (2001)	Perinatal (single case)	23	Jon’s recognition memory is selectively supported by familiarity. Difficulty appreciating the difference between *remembering* and *knowing*
Picard et al. (2013)	Perinatal 1 month	18, 19	No sense of recollection associated to bilateral hippocampal atrophy
Rosenbaum et al. (2011)	Perinatal (single case)	20	Greater deficit in recollection than in familiarity
**BRAIN TUMOR**			
Guillery-Girard et al. (2004)	3 and 6 (single cases)	10, 11	Ability to acquire novel semantic concepts despite profound episodic memory deficits
Martins et al. (2006)	6 and 5 (single cases)	9.5, 7	
Vicari et al. (2007)	3 (single case)	8	Profound episodic memory deficits with generally preserved semantic memory competencies
Svoboda et al. (2010)	13 (single case)	18	Severe episodic memory impairment with relatively well preserved general intellectual functioning
Pauly-Takacs et al. (2011, 2012)	11 (single case)	14	Profound episodic memory deficit in the context of well preserved premorbid semantic memory and new semantic learning. Lack of insight into own memory processes
**TRAUMATIC BRAIN INJURY**			
Hanten et al. (2000)	6	11	Verbal learning is within normal limits, but metacognitive predictions are less accurate compared to controls
Hanten et al. (2004)	7	12	Verbal learning and metacognitive monitoring are within normal limits, but ease-of-learning judgments are less accurate compared to controls
Crowther et al. (2011)	10	10–12	Verbal learning and metamemory poorer in children with severe TBI compared to milder forms of TBI and controls

#### Episodic memory studies

The amnesic syndrome is commonly understood to be a profound disorder of episodic memory in association with preserved or relatively preserved short-term memory and general intellectual abilities (Mayes, 1999). Episodic memory deficits can be caused by different factors such as lesions to the bilateral areas of the hippocampus as a result of anoxia, TBI, tumor, or cerebrovascular accident. The first reported case of amnesia with childhood onset is CC (Ostergaard, 1987). CC became amnesic following an anoxic episode at the age of 10 which resulted in multifocal brain damage also involving the hippocampal and parahippocampal regions. While his vocabulary acquisition was far from normal in a 5-year follow up test, he did show some progress suggesting that at least some residual learning took place in the absence of any measurable episodic memory. Broman et al. (1997) reported a 19-year follow up study of a boy who sustained focal hippocampal injury due to an anoxic episode at the age of 8 years; his brain injury severely compromised his episodic memory.

More recently, the episodic deficits in pediatric brain tumor patients have been explored. Brain tumors are the most common solid malignancies in childhood (Saran, 2002). Survivors of brain tumors often acquire complex cognitive difficulties including impairments in attention, processing speed, and different aspects of memory (Palmer et al., 2007). In particular, pediatric neuropsycho-oncology studies reveal clear episodic memory deficits (Guillery-Girard et al., 2004; Martins et al., 2006; Vicari et al., 2007; Svoboda et al., 2010). For example, CL developed severe anterograde amnesia following the surgical removal and subsequent chemo- and radiotherapy treatment of an ependymoma at the age of 4 years (Vicari et al., 2007). CL showed signs of significant memory difficulties in everyday life: she was not able to remember things she was asked to do and where she had put things. She had difficulties in reporting autobiographical events spanning from the previous few days to years. Similarly, CJ acquired a profound anterograde amnesia following treatment for a rare childhood brain tumor (germinoma) when he was 11 years old (Pauly-Takacs et al., 2011, 2012). CJ experienced particular difficulty retrieving context-rich episodic memories whether they had been encoded before or after the onset of his brain injury. In comparison, his premorbid general knowledge, vocabulary, and autobiographical information were remarkably well preserved. He was able to establish novel semantic facts in laboratory tasks and successfully updated the semantic component of his autobiographical memory in the 5-year period after diagnosis. A series of experiments demonstrated that CJ’s amnesia was characterized by a disproportionate deficit in source memory relative to item memory, whereby he permanently failed to accurately report the source or the context of a prior learning episode. Strikingly, his source memory deficit extended to encoding manipulations which enhance learning (e.g., self-generation by imagination). That is, although performance improved with self generation, CJ was no more able to accurately state that a correctly retrieved word had been in an imagination condition or not. This suggests that he was not consciously aware of the benefit in his performance, otherwise the logical response would be to adopt a bias, “if I remember this it must be because I self generated it earlier.” Further experiments suggested that CJ’s episodic retrieval was severely compromised by his inability to use source information as a basis for conscious recollection – even if this information was retained in memory (Pauly-Takacs, 2012). To date, studies exploring subjective states associated with memory have not yet been introduced to pediatric neuropsycho-oncology.

Vargha-Khadem et al. (2001) proposed the term developmental amnesia (DA) to describe cases who acquired focal bilateral hippocampal pathology very early in life following hypoxic-ischemic episodes (Vargha-Khadem et al., 1997; Gadian et al., 2000). These patients (as adolescents or young adults) presented with a dissociation between episodic and semantic memory. While they had a profound impairment in remembering daily life events, they attended mainstream education where they acquired literacy skills as well as normal levels of intelligence and knowledge. DA is particularly interesting because, as opposed to adult-onset amnesia, there is no well-established prior memory competence that is subsequently lost as a result of a specific brain insult. This led to strong theoretical claims about the functional and neural organization of declarative memory during development. Based on cases of DA, a neuroanatomical model of declarative memory was proposed which postulates that the development of semantic memory depends on parahippocampal cortices but not on the hippocampus, while episodic memory development largely depends on the hippocampus (Vargha-Khadem et al., 1997; Gadian et al., 2000).

In the original description of DA by Vargha-Khadem et al. (2001), the authors suggest a distinction between recollection-based versus familiarity-based judgments. However, studies exploring whether or not children with DA can recall detailed episodic memories and also introspect on these memories are extremely rare. To date, most of the evidence comes from a widely studied case of DA, Patient Jon, who became amnesic as a result of perinatal anoxic episode. The available evidence seems to converge on the conclusion that Jon’s recognition memory is selectively supported by familiarity (Baddeley et al., 2001). While Jon readily assigned R responses to a good proportion of correctly recognized items, his justifications for them did not reflect true recollective experience; rather, it appeared that Jon gave “R” judgments when he experienced a sense of ease of access to the items (i.e., fluency as opposed to contextual retrieval) which gave rise to higher confidence in his memory (Baddeley et al., 2001; Gardiner et al., 2008). It was also shown that Jon’s recognition memory performance fell significantly below that of controls in tasks where recognition is usually facilitated by recollection specifically (e.g., deeper processing, task-enactment) (Düzel et al., 2001; Gardiner et al., 2008). Finally, at the physiological level, it has been demonstrated that Jon lacks the event-related potential (ERP) index of recollection.

Consistent with the above, a recent assessment employing the Process Dissociation Procedure (PDP; Jacoby, 1991) found that Jon’s recognition memory is characterized by intact familiarity but severely compromised recollection (Brandt et al., 2009). In this paradigm, participants complete two different memory tasks, one where they merely have to identify whether or not they have studied the item before, and one where, additionally, they have to specifically declare where or when the item was studied. By comparing performance on the test assessing specifics (which requires recollection) and the test asking for a simple old/new distinction (which merely requires familiarity) it is possible to estimate the separate contributions of familiarity and recollection.

More recently, Picard et al. (2013) explored recollection as measured by the RK paradigm in two cases of DA (Valentine and Jocelyne, both adults who suffered from brain injuries that led to bilateral atrophy of the hippocampus). Both patients’ episodic memory was first assessed using the House test (Picard et al., 2012), an ecological test designed to assess what-where-when features coupled with RK judgments. On this task, both patients showed major difficulties, with spatial and temporal context recall at floor. For the RK judgments, Valentine was unable to justify her R responses and Jocelyne could not grasp the difference between Remember and Know judgments, despite clear explanations. Similar difficulties were found when an autobiographical memory was used (TEMPau task, Piolino et al., 2009). Furthermore, the two patients recalled fewer specific autobiographical memories than controls with a clear lack of episodic details (for similar findings, see Kwan et al., 2010). In general, these studies point to a specific recollection deficit in DA. However, the recollection/familiarity dissociation may not always be as clear-cut. Using the R/K paradigm and the ROC procedure, Rosenbaum et al. (2011) reported a case in which *both* recollection and familiarity appeared to be impaired, although there was a trend toward greater deficit in recollection.

#### Metamemory studies

Of the different etiologies of childhood neuropathology reviewed in this section, children with TBI are the only population where metamemory research using contemporary experimental paradigms has already begun. Considering that axonal injury and focal lesions to frontal cortical areas of the brain are the most common forms of pathology following childhood TBI (Tong et al., 2004), it is probable that disruption to frontally guided networks such as those supporting recollection and metamemory will be impaired. To the best of our knowledge, only three experimental studies using metamemory judgments in children with TBI have been reported, all by the same research group. Hanten et al. (2000) measured children’s metacognitive awareness during a multi-trial verbal learning task. In this study, a small group of children with a mixed level of TBI severity (see Table 1) and an age-matched control group were asked to make an Ease of Learning (EOL) and a JOL prediction with respect to their recall performance after a 2-h delay. Contrary to previous reports, Hanten et al., did not find significant differences in recall performance between the TBI and the control group either across the four learning trials or after the delay. However, significant differences were found with respect to the two metamemory measures, such that children with TBI were less accurate at judging the ease with which an item would be learned (EOL) as well as at predicting recall of an item (JOL). In another study, Hanten et al. (2004) tested 37 children with severe and 40 children with mild TBI, and compared their performance to an age-matched control group in the same verbal learning paradigm. Similarly to that found by Hanten et al. (2000), learning and forgetting rates did not differentiate between the groups. With respect to metamemory, all groups tended to overestimate their future recall performance, but children with TBI (i.e., irrespective of severity) did so to a significantly greater degree. Furthermore, the severe group had particular difficulty accurately predicting their performance on the EOL measure. By contrast, no differences were found between the three groups on JOLs suggesting that after gaining experience of the learning task, children with TBI were as able as typically developing children to monitor their learning.

In an attempt to elucidate recovery of memory and metamemory function following childhood TBI, Crowther et al. (2011) classified children with TBI into mild, moderate, and severe groups and considered memory and metamemory performance in a multi-trial verbal learning task across five assessments over a 2 year period. The results indicated that children with moderate and severe TBI showed the greatest improvement across all measures over time, but the performance gap between them and the mild TBI group increased. Contrary to some of the earlier studies with smaller samples, brain injury severity did affect levels of learning across trials in this study. TBI severity was also associated with poorer JOL accuracy.

### Autism

Autism is a NDD that primarily affects social interaction and communication (American Psychiatric Association, 1994). In this review we present studies including adults and children and report all studies in the Table 2.

**Table 2:** **Key findings on episodic memory and metamemory in Autism**.

Reference	Age (years)	Main finding
Boucher and Warrington (1976)	9 (Exp. 1), 13 (Exp. 2), 12 (Exp. 3), 10 (Exp. 4), 14 (Exp. 5)	Impaired recall and recognition and spared cued recall in ASD
Lind and Bowler (2009)	8	Spared performance on a delayed self-recognition task
Bowler et al. (2007)	33 (Exp1), 33 (Exp. 2), 35 (Exp. 3)	Fewer Remember responses in Asperger syndrome
Gras-Vincendon et al. (2007)	24	Spared memory for temporal context
O’Shea et al. (2005)	10	Impaired source memory for social context
Salmond et al. (2005)	12	Selective episodic memory impairment and preserved semantic memory
Souchay et al. (2013)	14	Spared recollection as tested by objective measures (source memory tasks), but relatively impaired recollection as tested by subjective measures (R/K paradigm)
Bowler et al. (2000a)	29 (Exp. 1 and 2)	Comparable to typical performance on illusory memory task, but memory characterized by a *know*-like rather than *remember*-like profile
Bennetto et al. (1996)	16	Impaired temporal order memory
Tanweer et al. (2010)	34	Poorer recall of AM in Asperger syndrome (AS). Personal memories recalled related to *K* rather than *R* responses
Wilkinson et al. (2010)	13	Inaccurate metacognitive judgments-of-confidence in a face recognition task in children with ASD
Wojcik et al. (2011)	11	Accurate judgment of confidence on a self-performed action task
Wojcik et al. (2013b)	12 (Exp. 1), 13 (Exp. 2)	Spared immediate and delayed judgment-of-learning
Wojcik et al. (2013a)	12	Impaired episodic FOK, spared semantic FOK

#### Episodic memory studies

The “developmental disconnection model” links the symptoms of autism spectrum disorder (ASD) to weak functional connectivity in the brain (Belmonte et al., 2004), and neuroimaging suggests that, for example, deficient self-reflective thought processes (e.g., Theory of Mind) are directly linked to brain abnormalities. A growing literature has also identified memory impairment in autism. Most studies have explored memory in adults with high-functioning autism or Asperger syndrome. Word-pair association learning has been used widely in autism to assess episodic memory (Boucher and Warrington, 1976). Numerous studies have shown that performance on recognition and cued-recall tasks is mostly unimpaired in autism (see Boucher et al., 2012 for a recent review). In fact, research into ASD has only revealed subtle impairments in episodic memory, particularly on free recall tasks of semantically related word lists (see Boucher et al., 2012). Furthermore, no deficits have been reported on semantic memory tasks (Salmond et al., 2005; Bowler et al., 2007; Lind and Bowler, 2009; Wojcik et al., 2013a). Several studies suggest that the memory profile observed in ASD is again linked to abnormalities of the hippocampal formation and other neural regions including the amygdala and the frontal cortex (Boucher et al., 2005; Salmond et al., 2005).

The recollection of children with ASD has been examined with source memory tasks, which might point to a deficit in the retrieval of specifics; an idea which is congruent with the notion that higher order and self-reflective processes are impaired in ASD. Only a very few studies have explored the detail retrieved in ASD (see Bon et al., 2012). The studies report conflicting findings. For example, in a first study, Bennetto et al. (1996) used temporal intrusions as a measure of source memory. Participants (adolescents with ASD and TD controls) were given the California Verbal Learning Test (CVLT; Delis et al., 1987) and the number of intrusions of items from previous lists was measured. It was found that the responses of adolescents with ASD included more intrusions than responses of controls. Participants with ASD also had lower performance when asked to indicate which items had been presented most recently. Using a similar recency task with picture stimuli, Gras-Vincendon et al. (2007) explored this further and found no deficit in adults with ASD. However, Bigham et al. (2010) demonstrated recency judgment impairments in low functioning children/adolescents with ASD. In a more recent study (detailed below), Souchay et al. (2013) showed that adolescents with ASD could recall different types of source information correctly (voice, color, spatial, and temporal localization). However, other studies have suggested that source memory might be more impaired in ASD when the contextual information involves a social aspect, as impairments in social functioning are a primary characteristic of ASD (O’Shea et al., 2005). In other words, it could be that contextual information of the social type is particularly impaired in ASD, mirroring the more fundamentally social nature of this disorder. Furthermore, the recall of contextual information can sometimes be spared alongside an impairment in subjective experience, thus revealing a fractionation of recollection itself. In autism these findings fit well with the brain abnormalities reported in the activation of the default-mode network in autism, and possibly point toward the importance of the self in episodic memory and metamemory functioning (Klein, 2013).

Turning to the RK paradigm, adults with Asperger’s syndrome have consistently shown a reduced number of Remember judgments (Bowler et al., 2000a,b, 2007; Tanweer et al., 2010). In a recent study, Souchay et al. (2013) explored subjective states associated with episodic retrieval in adolescents with ASD. The novelty of this study was to measure recollection using objective and subjective methods in the same task. In three different experiments, the same group of participants was presented with information to learn in a specific context (color and voice in Experiment 1, temporal context in Experiment 2, and spatial context in Experiment 3). At the recognition stage, all participants reported R or K (subjective measure). Furthermore, after the recognition task, they performed a source memory task (objective measure) in which all items presented at encoding were re-presented and participants were asked to recall the source. All three experiments showed that adolescents with ASD could, like Typically Developing controls, correctly recall source information. This suggests that recollection, as measured by the retrieval of contextual information, is preserved in adolescents with ASD. However, recollection as measured by the subjective report, was shown to be impaired, at least in Experiment 1, where the ASD group gave significantly fewer Remember responses than controls. These findings point to a specific and subtle recollection impairment in adolescents with ASD. Souchay et al. (2013) propose that memory in autism, and in particular recollective experience, might be characterized by a lack of rich details related to the self. This interpretation fits with the resting state connectivity studies in autism showing abnormalities of the default-mode network including: the ventral medial prefrontal cortex, the posterior cingulate/retrosplenial cortex, inferior parietal lobule, lateral temporal cortex, dorsal medial prefrontal cortex, and hippocampal formation (DMN) (Buckner et al., 2008). Several studies have now shown that the DMN plays a putative role in self-referential representations (Buckner et al., 2008), and the implication is that although source information is accessible in ASD it does not give rise to the same experience of remembering, nor is not bound into a whole representation which is re-activated by re-experiencing the event in the past.

#### Metamemory studies

**Figure 2:**
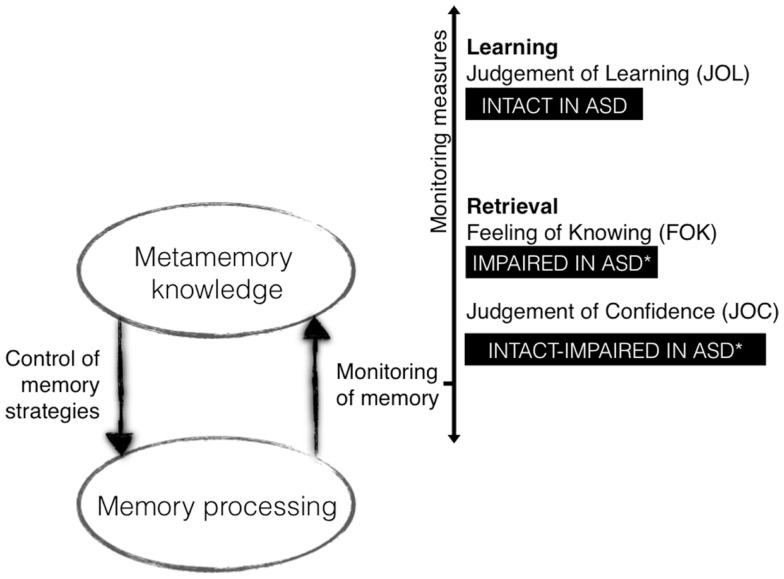
**Metamemory findings in autism spectrum disorder**.

In autism, studies exploring whether or not children, adolescents, or even adults can reflect metacognitively on their memory and their episodic contents are scarce (see Figure 2 for a summary). Some research has considered memory confidence, and produced equivocal findings. These judgments-of-Confidence (JOCs), consist of asking participants to estimate the correctness of their answer once they have produced it. Some research points to a deficit in this capacity. For instance, Wilkinson et al. (2010) investigated JOC in children with ASD using a face recognition task – an ability thought to be impaired in autism (e.g., Rouse et al., 2004; Molesworth et al., 2005). After studying 24 face photographs participants carried out a recognition test in which they were presented with 48 photographs and had to recognize the faces presented earlier. Children gave JOCs by reporting whether they were “certain,” “somewhat certain,” or “guessing” their answers. Memory performance did not vary with the levels of certainty in the ASD group. In short, children with autism did not discriminate between correct and incorrect responses in their confidence judgments. However, Wojcik et al. (2011) showed that children and adolescents with ASD made as accurate JOCs as controls when asked to predict their recall of school-like instructions (e.g., pick up the red ruler and put it in the yellow box, then touch the blue pencil). Thus, studies exploring accuracy of judgments made on information retrieved have revealed contradictory findings, but the extent to which this might relate to the materials in question, or sample differences is not known.

To explore metamemory during encoding, Wojcik et al. (2013b) explored accuracy of JOLs predictions. The aims of this experiment were to see whether participants with ASD would make accurate JOL predictions and also whether JOL accuracy would vary with the time at which JOLs are elicited. Both healthy children (Schneider et al., 2000; Koriat and Shitzer-Reichert, 2002) and adults (Nelson and Dunlosky, 1991) show that delayed JOLs (made some time after study) are far more accurate than immediate JOLs (made immediately after study); this is a robust *delayed JOL effect*. According to Dunlosky and Nelson (1992), this effect occurs because the delayed JOL is based on information which in fact resembles the information retrieved from long-term memory when searching for the target word; thus the effect pertains to the monitoring of episodic processes during retrieval. Wojcik et al. (2013b) presented children with ASD with two separate immediate and delayed JOL tasks. They presented two sets of 24 word pairs at study (e.g., dog-CAR). For the immediate JOL task, after studying each pair they were re-presented with the cue word and asked to make a JOL by predicting whether in about 5 min they would be able to recall the target word when shown the cue word. In the delayed condition, JOLs were given in the same way but after the study of all pairs. The results showed that children with ASD could accurately predict their later recall with both types of JOL, and critically, like TD children they could switch to using more appropriate mnemonic cues. Such an effect arguably demonstrates an ability to use retrieval from memory in order to accurately gage future memory performance, a self-reflective capacity. Finally, Wojcik et al. (2013a) used the episodic FOK (Souchay et al., 2000) paradigm to explore self-reflecting processes at the retrieval stage. Children with ASD were found to give inaccurate episodic FOK judgments in comparison to typical children, suggesting impairment in estimating content at retrieval when information is not easily accessible (the same study showed that semantic FOK was unimpaired).

There are thus conflicting findings in the autism literature, which we have posited might be influenced by the type of information required to make the metamemory judgments. People with ASD may be particularly impaired at the type of reflection captured in feelings of remembering, which we have argued elsewhere are particularly necessary for producing accurate episodic FOK judgments (Souchay et al., 2007; Wojcik et al., 2013a). Of particular interest, the brain network engaged in metamemory (Kikyo et al., 2002; Maril et al., 2003; Kikyo and Miyashita, 2004; Schnyer et al., 2005) and recollection (Yonelinas, 2002; Eichenbaum et al., 2007) shows some overlap with the brain network found underactive in autism. Thus, a decrease in functional activity of these brain networks could lead to some metamemory judgments being inaccurate, such as episodic FOK judgments, and this would presumably have consequences for the regulation and higher order control of episodic memory.

### Williams syndrome, Down syndrome, and 22q11.2 deletion syndrome

A recent development in the scientific literature consists of exploring cognition in genetic disorders. Amongst the most explored disorders in children are Williams syndrome (WS), DS, and 22q11.2 deletion syndrome (22q11DS). However, despite the recent growth in this research field, studies exploring episodic memory and metamemory are again scarce in these populations. Furthermore, interpreting the deficits observed relies on our current knowledge of the neural circuits involved in the normal functioning of the memory and the maturation of those specific circuits in these genetic disorders. This section presents a summary of the few studies exploring recollection and subjective states of episodic memory in WS, DS, and 22q11DS and an attempt will be made to link with brain functioning. We report these findings in Table 3.

**Table 3:** **Key findings on episodic memory and metamemory in children and adolescents with genetic disorders**.

Article/etiology	Age (years)	Main finding
**WILLIAMS SYNDROME**		
Edgin et al. (2010)	18	Deficits in spatial and verbal associative memory compared to individuals with Down syndrome
Costanzo et al. (2013)	20	Reduced recollection and spared familiarity on the process dissociation procedure and task dissociation procedure
Jarrold et al. (2007)	18	Poor performance on long-term memory for visual information
Vicari et al. (1996)	10	Long-term memory deficit on verbal and visual tasks
**DOWN SYNDROME**		
Edgin et al. (2010)	17	Deficit in associative memory
Carlesimo et al. (1997)	16	Deficit in explicit memory, difficulties in organizing material at encoding, retrieval deficits
Jarrold et al. (2007)	14	Poor performance on long-term memory for visual information
**22q11.2 DELETION SYNDROME**		
Debbané et al. (2008a)	17	Inefficient recollection-based retrieval, failure to correctly identify contextual information
Debbané et al. (2008b)	14	More source errors, more confusions between exterior sources in which the self was not involved

#### Williams syndrome

Williams syndrome is a relatively rare NDD (prevalence of 1 in 20,000 births) with confirmed genetic origin (random deletion of approximately 25 genes on chromosome 7, specifically 7q11.23; Ewart et al., 1993). Recently this disorder has attracted a great deal of attention due to the unique aspects of social, behavioral, and cognitive deficits. The cognitive profile in WS is characterized by deficits in executive functioning (Rhodes et al., 2010; Carney et al., 2013) and many studies assessing working memory have now demonstrated a dissociation between relatively proficient skills within the verbal domain and more severe impairments associated with visuo-spatial processing (Hoffman et al., 2003; Vicari et al., 2003).

In WS, studies exploring episodic memory are in their infancy. Long-term memory studies have confirmed the dissociation between verbal and visual tasks (Vicari et al., 1996; Edgin et al., 2010). Furthermore, several studies have compared performance in WS in recall and recognition tasks. Jarrold et al. (2007) showed that recall was more impaired than recognition, although Vicari (2001) reported preserved free recall and recognition of verbal and visual information. It is generally accepted that familiarity has a significant role in recognition but not in free recall, which might point to a deficit in recollection (in recall) but not familiarity (in recognition). However, the conflicting findings in WS regarding recall and recognition are inconclusive. To clarify this issue, Costanzo et al. (2013) explored recollection in WS using two paradigms (PDP and associative recognition) to measure recollection objectively. Results of the two experimental tasks showed a reduced contribution of recollection and a preserved contribution of familiarity. Indeed, the exclusion condition of the PDP task demonstrated that children with WS failed to remember the modality in which the items had been presented whereas the associative recognition task showed that children with WS failed to identify the visual stimulus that had been associated with a target at encoding. According to the authors, these findings suggest that maturation of memory abilities in children with WS is not globally delayed but that instead it shows qualitatively different developmental trajectories. This parallels the observations reported by Meyer-Lindenberg et al. (2005) that the hippocampus in WS shows differences in shape and functioning (reduced resting blood flow in the anterior portion of the hippocampus); deficient hippocampal maturation could potentially underlie poor recollection in WS. Finally, to the best of our knowledge, no study has yet explored subjective states either those associated with retrieval in WS, or the ability to estimate memory as measured by metamemory judgments.

#### Down syndrome

Down syndrome affects about 1 in 1000 live births (Sherman et al., 2007) and is caused by abnormalities of chromosome 21. In the DS cognitive profile, working memory is typically impaired. People with DS have a deficit in verbal working memory, with non-verbal working memory comparatively preserved, presenting a double dissociation with WS (Baddeley and Jarrold, 2007). More recently, Lanfranchi et al. (2012) showed executive impairment in people with DS. In contrast with multiple studies exploring working memory, only a few studies have explored episodic memory in DS. Carlesimo et al. (1997) showed reduced free recall and reduced recognition in DS. More recently, Edgin et al. (2010) showed reduced associative memory in adolescents with DS on two tasks: a word list learning task (NEPSY list learning test, Korkman et al., 1998) and the spatial Paired Associate Learning (PAL) task from the CANTAB (Sahakian and Owen, 1992). To the best of our knowledge, no study has yet explored recollection, subjective states of episodic memory or metamemory in DS (and this clearly remains a priority). However, a recent single case study exploring autobiographical memory in a 22-year-old male with DS, patient PQ, points to an impairment in recalling detailed memories in DS. Patient PQ’s autobiographical memory was characterized by a significantly impoverished recall of specific memories (Robinson and Temple, 2010). Volumetric MRI studies indicate that people with DS have smaller volumes in temporal areas including the hippocampus (Schmidt-Sidor et al., 1990); suggesting that recollection and subjective states of episodic memory might be deficient in DS, but this area still remains to be explored.

#### 22q11.2 deletion syndrome

22q11.2 Deletion syndrome, also known as velo-cardio-facial syndrome (VCFS) is a genetic disorder associated with a microdeletion in chromosome 22q11, estimated to occur in one of every 6000 live births (Botto et al., 2003). Behaviorally, people with 22q11DS also often present with schizophrenia or attention deficit/hyperactivity disorder (ADHD) (see Debbané et al., 2006). A number of studies have explored structural brain differences in 22q11.2DS and these usually report that brain volume in children is between 8 and 11% smaller than controls. Most neuroimaging studies report a total hippocampal volume reduction (Debbané et al., 2006; Kates et al., 2006; Deboer et al., 2007; Flahault et al., 2012). According to Flahault et al. (2012) this reduction of volume could be partly due to a reduction in the amount of input received from connected cortical regions such as the parieto-lateral cortex, the posterior cingulate, and the temporal cortical structures, known also to be reduced in 22q11.2DS.

Little is known about the cognitive characteristics of this syndrome. So far, studies have mainly assessed executive functioning and all report that people with the 22q11DS have marked impairment in visual attention and executive function (Sobin et al., 2004, 2005). A recent study also showed that differences in brain activation (parietal and occipital regions) explained deficits on a visuo-spatial working memory task (Azuma et al., 2009), thus suggesting that differences in the development of specific brain structures might underpin the cognitive deficits in this population.

Studies exploring episodic memory suggest that people with 22q11.2DS (adolescents and adults) demonstrate similar levels of recognition performance for materials such as words, pictures, or even action statements (Debbané et al., 2008a,b). However, Debbané et al. (2008a) showed in two experiments deficient retrieval skills in adolescents and adults with 22q11.2DS (ages 10–36 years old). In Experiment 1, a directed forgetting paradigm was used (e.g., Bjork, 1970), where participants were instructed to control their encoding according to whether the items were presented as “to be remembered” or “to be forgotten.” In Experiment 2, a continuous recognition task was used, in which participants were asked to identify pictures that appeared twice within a list of picture items. In this task, several different lists were used. Participants were instructed to detect repetitions within the list only; items presented in previous lists were to be responded to as novel. In Experiment 1, people with 22q11.2DS were found to produce more false alarms and in Experiment 2 they were found to have more commission errors (higher tendency to incorrectly classify previous list distractors as within list repetitions). The results of Experiment 2 are of particular interest as they suggest that people with 22q11.2DS have specific difficulties in using contextual information (in this case temporal information: current versus past list, paradigm adapted from Schnider and Ptak, 1999) to correctly reject items. According to the authors, these errors might be due to inefficient binding between target and temporal context information and therefore could also point to diminished recollection processes in 22q11.2DS. Deficits in contextual information were also reported by Debbané et al. (2008b) in a source monitoring task in which patients were given action statements and were asked to imagine themselves performing the action or to imagine the experimenter performing the action. Results showed that adolescents with 22q11.2DS committed more source confusion errors than controls. Altogether, these findings thus suggest that recollection, at least as assessed by objective measures (such as source) is impaired in 22q11.2DS.

To the best of our knowledge, no study has yet explored subjective states of episodic memory either using the RK paradigm or metamemory judgment paradigms. However, subjective states of episodic memory as measured by RK are impaired in schizophrenia in that patients with schizophrenia report fewer R responses (Libby et al., 2013) and metamemory has also found to be inaccurate in schizophrenia (Souchay et al., 2006). Therefore, given the behavioral similarities observed with people with 22Q11.2, it seems reasonable to predict impairments in these subjective states or evaluation of memory contents. This remains a priority for future research.

## Discussion

The purpose of this article was to review the subjective states associated with episodic retrieval from a neurodevelopmental perspective. This review supports the idea of a fractionation of the episodic memory system and clearly suggests that episodic memory is a multifaceted system as suggested by many theories (Tulving, 2002; Montaldi and Mayes, 2010; Klein, 2013). Within these theories, memory contents are experienced as episodic only if certain operations occur at retrieval, such as retrieval of contextual information or reflective and self-referential processes. This review presented some examples within the developmental literature of dissociations between such processes or different developmental trajectories.

### Episodic memory in typically developing children

The first part of this review (see Typically Developing Children), highlighted a developmental change in episodic memory in typically developing children. It is possible to measure separable contributions of a more automatic, perceptual memory system (i.e., familiarity) and a more conceptual higher order system (such as recollection) even at a young age. Clearly however, there are limitations of the episodic memory system in early life. For example, recollection and familiarity are dissociable at about 5 years (Riggins et al., 2013), but recollection processes continue to develop until adulthood. Of particular interest, different developmental trajectories between gist and recollection are found when recollection is measured objectively by contextual details or subjectively by first-person experiences of remembering. As they get older, children report more and more experiences of remembering.

Similarly, increasing evidence suggests that even young children have insight into their memory contents (see Lyons and Ghetti, 2010). Thus, as children develop, they can form and retrieve richer episodic memories and evaluate the content of these memories to guide their learning. This increase in experience of remembering and the formation of rich contextual memories presumably impacts on the development of metamemory. Indeed, a development in the metacognition literature suggests that recollection is used to guide metacognitive judgments (Hicks and Marsh, 2002; Souchay et al., 2007); predictions of future performance are more accurate when contextual information can be retrieved and when there is the feeling of “remembering.” We propose then, that the development of richer episodic memories influences the metacognitive assessment of memory content.

Finally, the developmental changes in episodic memory occur alongside the maturation of the brain (see Ghetti and Bunge, 2012; Ofen, 2012 for review). Structural and functional imaging studies suggest a neural specialization with increasing age, such as for example the developmental shift from posterior to anterior hippocampus (see Figure 2). The few studies published so far linking brain developmental changes and memory suggest that this brain specialization parallels the development of episodic memory and the development of recollection. The complex network of temporo-frontal structures critical for episodic memory function in healthy adults, and implicated in memory and metamemory disorder in brain-injured populations, include some of the last brain structures to mature. The maturation of brain regions referring to the social brain and CMS has to be taken into consideration when examining the development of subjective recollection. The few studies using social-cognition tasks report an hyperactivation of the CMS in children compared to adults. The same phenomenon is observed in studies of mental-state attribution (Blakemore, 2008). These authors suggested that this decrease in activity from adolescence to adulthood would be related to an immature or undefined self-appraisal. To better determine how each of these brain structures intersects with the development of the different episodic components, further longitudinal studies are needed. For example, to the best of our knowledge, no study has yet explored the development of metacognitive judgments in relation to neural specialization in children.

### Episodic memory in neurodevelopmental disorders

The second part of this review (see Neurodevelopmental Disorders) addressed episodic memory in different NDDs including acquired amnesia, autism, and genetic disorders. This review shows that this strand of research is still in its infancy and that most studies exploring subjective states of episodic memory have been done in children with acquired amnesia and autism whilst such studies are rare in genetic disorders. Despite the novelty of this type of research, the main outcome of these studies is to show that across a broad set of different NDDs there are various types of episodic memory impairment, each with possibly a different character. This literature is thus in agreement with the idea that episodic memory is a multifaceted process (Klein et al., 2004) and that therefore fractionations or dissociations might occur.

Clearly, a child with autism does not present with the same kind of memory deficit as a child with an acquired head injury, but nonetheless they may fail the same episodic memory task, albeit for different reasons. For example, the literature presented here suggests that acquired amnesia is characterized by source memory deficits, whilst in ASD these source memory deficits might be specific to the self. If we can understand the separate contributions to episodic memory impairment implicated in each clinical group in correspondence with their brain damage, we should be able to determine whether a specific brain lesion can affect the development of an episodic component in particular, or if such a lesion will have broader consequences in terms of episodic memory. We will also be better able to compensate for failing mechanisms, and emphasize intact abilities (as has been suggested as an outcome of the metacognition research in autism, for example (Wojcik et al., 2013a).

As an example, consider that Souchay et al. (2013) present a case where a group of people with autism can successfully report the source of an item, but yet, this does not seem to be captured in their subjective experience of remembering. The objective information drawn from recollection, does not seem to be phenomenologically re-experienced in the same way as in controls. In other words, the capacity to retrieve episodic content (source information) could be dissociated from the ability to introspect on the memory content (R responses). Similarly, Wojcik et al. (2013a) showed that a capacity to retrieve memory content (cued recall) was associated with inaccurate introspection (FOK judgments). Interestingly, the dissociations observed in ASD, in some ways resonate with the case of patient R.B. summarized in Klein (2013) presenting a dissociation between memory content (source) and sense of ownership. Here then, we attempt a fractionation of the recollection processes critical to episodic memory function. To attempt such a fractionation, we will have to see episodic memory dysfunction as something rather more nebulous and complex than forgetting (Klein et al., 2004). Recent work suggests that metacognitive judgments and agency judgments are interrelated and thus capture similar processes (Cosentino et al., 2011). Such a relationship would be worth exploring in NDDs to examine this fractionation of episodic memory.

We suggest that to achieve a better understanding of how episodic memory and subjective states of episodic memory develop- and the brain regions involved-further investigations are needed. For example, studies in NDDs could start by exploring whether or not children with NDDs understand the difference between “remembering” and “knowing.” Indeed, Jon’s case has shown that individuals who grow up without a functional episodic memory system may not be able to appreciate the difference between “remembering” and “knowing.” This concern that amnesic patients may not be able to reliably introspect about their own memory processes has been raised in the adult literature as well (Turriziani et al., 2008). This contrasts with the studies in the metamemory literature showing that amnesic patients can predict their memory performance accurately (Janowsky et al., 1989). That is, a fractionation could occur between different subjective states or different ways of introspecting. Introspection (or cognitive monitoring) is crucial to guide strategic behavior (Nelson and Narens, 1990). Thus, other forms of introspection could be explored in children with NDDs, and especially those involved in early strategic behavior such as uncertainty judgments (Lyons and Ghetti, 2013). Neurodevelopmental studies could also assess sense of ownership, agency, and authorship to see if these dissociate from the memory content (see Synofzik et al., 2008 for a theoretical framework to explore agency and ownership). In this context, how the self develops in relationship to episodic memory could be considered through ownership. Indeed, van den Bos et al. (2010) have shown that when participants were asked to sort items into baskets, a self-ownership effect was found on recognition and more for Remember than Know responses. Furthermore, in a more recent study, Cunningham et al. (2013) found that this effect could be used with very young children, thus showing how the self shape memory performance. Such paradigms could be of particular interest to explore in NDDs. Finally, the quality of the episodic contents recalled should also be examined. For example, in adults with Asperger’s syndrome, Bowler et al. (2007) showed that the quality of the justifications given to R responses were similar to the ones given by controls, suggesting a difference in quantity and not quality. This issue is particularly important from a developmental perspective and for example in neurogenetic disorders. Do the memory processes just develop later and/or in a qualitatively different manner? Such studies could help us to determine whether or not the content of a memory and the operations occurring at retrieval can be dissociated.

To conclude, we have shown that different episodic processes develop at different rates, and can be dissociated in different clinical groups. In fact it is relatively early in the field to make any strong claims, except that subjective experiences and strategic factors are both critical in the regulation of memory and should therefore be explored further in NDDs. Indeed, where one or other of these processes is impaired or delayed developmentally, there will be consequences in the cognitive system which will be of interest to clinicians and educators working with individuals with neurodevelopmentally disordered groups.

## Conflict of Interest Statement

The authors declare that the research was conducted in the absence of any commercial or financial relationships that could be construed as a potential conflict of interest.
